# Comparative analysis of machine-learning methods for prediction of pilot performance during startle events from neuropsychophysiological features of stress resilience and cognitive task scores

**DOI:** 10.3389/fphys.2026.1879388

**Published:** 2026-07-10

**Authors:** Mate Gambiraža, Siniša Popović, Matej Kurtak, Krešimir Ćosić

**Affiliations:** University of Zagreb Faculty of Electrical Engineering and Computing, Zagreb, Croatia

**Keywords:** acoustic startle reflex, cognitive performance, feature selection, flight simulator, machine learning, pilot performance prediction, stress resilience

## Abstract

**Introduction:**

Unexpected startle events can impair pilot perception, decision-making, and performance during time-critical flight operations, making objective predictors of individual vulnerability relevant to next-generation air combat.

**Methods:**

Fifteen military pilot cadets completed a two-phase protocol in which context-free laboratory paradigms assessed neuropsychophysiological stress-resilience features and cognitive task scores, followed by a flight-simulator task involving unexpected perturbations. Pitch-tracking error was predicted using multiple regression and machine-learning regression models evaluated with leave-one-out cross-validation, with preprocessing and feature selection performed within the training folds.

**Results:**

The baseline multiple-regression model achieved a mean absolute error of 2.55° and a correlation of approximately 0.70. Under the fixed domain-balanced feature strategy, the regression neural network achieved the best predictive accuracy, with a mean absolute error of approximately 2.07° and a correlation of approximately 0.75. However, improvement over the interpretable baseline was modest, and performance depended more on feature-selection strategy than on model type. Domain-level ablation indicated that startle-related and allostatic features contributed most to prediction.

**Discussion:**

These findings suggest that, for small and heterogeneous pilot datasets, research-guided feature selection and explainable models remain highly competitive, with limited practical benefit from more complex nonlinear approaches.

## 1 Introduction

The operational environment of contemporary military aviation places increasingly complex demands on the human operator. In fifth- and sixth-generation combat aircraft, pilots must maintain effective performance under conditions of high workload, rapid maneuvering, compressed decision timelines, dense information streams, and complex interaction with advanced onboard systems. Under such conditions, pilot performance depends not only on technical proficiency, but also on the ability to regulate physiological arousal, sustain cognitive control, and adapt rapidly to unexpected events. Although avionics, automation, and decision-support systems have advanced substantially, human factors remain a major source of risk in a modern aviation, and pilot states such as high workload, fatigue, stress, reduced situational awareness, and startle effect continue to be associated with performance degradation and flight-safety threats (Borghini et al., 2014; Diarra et al., 2023; Kyle et al., 2025; Landman et al., 2017; Li et al., 2008; Pütz et al., 2024; Rankin, 2007; Van Weelden et al., 2022; Wiegmann and Shappell, 2003). Current pilot selection and evaluation procedures provide valuable information on cognitive ability and behavioral performance, but they still tend to underrepresent physiological and stress-resilience characteristics that may become critical under sudden or extreme conditions (Ćosić et al., 2019a, b; Flood and Keegan, 2022; Jones and Endsley, 1996; Miyatsu et al., 2023; Šarlija et al., 2021; Vine et al., 2015).

Among the acute factors that challenge pilot functioning, startle events have operational relevance. Startle response is a rapid involuntary physiological, cognitive, and behavioral response to sudden, intense, or threatening stimuli, and in aviation it has been associated with disrupted attention, degraded situational awareness, delayed decision-making, and reduced control precision during critical flight phases (Ćosić et al., 2016; Diarra et al., 2023; Kinney and O’Hare, 2020; Landman et al., 2017; Martin et al., 2015; Rivera et al., 2014). Although fundamentally as a defensive response, startle reaction can transiently impair working memory, vigilance, problem solving, and motor coordination, especially when combined with concurrent distraction, uncertainty, or high workload (Kinney and O’Hare, 2020; Martin et al., 2015; Talone et al., 2015). Recent simulator studies continue to support the operational significance of unexpected startle events such as high-urgency events that have been shown to impair pilot trainees’ flight performance, while stronger startle and surprise responses have been associated with poorer information-processing performance during simulated in-flight events (Chen et al., 2026; Peng et al., 2025). These findings reinforce the importance of understanding individual neurobiological differences regarding startle vulnerability and recovery when studying pilot performance under time-critical conditions.

A growing body of research suggests that resilience to acute stress cannot be captured adequately only by behavioral observation or conventional psychological testing. Individual differences in resting autonomic regulation, defensive behavioral reactivity, and physiological recovery appear to influence how effectively operators maintain performance under pressure (Ćosić et al., 2019b; Diaz-Ramos et al., 2021; Miyatsu et al., 2023; Šarlija et al., 2021; Walker et al., 2017). Resting autonomic indices such as heart rate variability (HRV) and respiratory sinus arrhythmia (RSA) have been associated with regulation flexibility and executive control, whereas acoustic startle characteristics reflect defensive behavioral reactivity, habituation, and stress vulnerability (Bradford et al., 2014; Carnevali et al., 2018; Pole et al., 2009; Risbrough and Davis, 2013; Walker et al., 2017). Measures of allostatic reaction and recovery provide complementary information about how efficiently physiological balance is restored after perturbation (Carnevali et al., 2018; Diaz-Ramos et al., 2021; Hourani et al., 2020; Walker et al., 2017). In parallel, cognitive capacities such as attentional control, working memory capacity, cognitive control, and multitasking abilities are central to effective performance in military aviation and can be assessed with standardized tasks such as the n-back, paced auditory serial addition task (PASAT), multiple-object tracking, and dual Stroop paradigms (Barron and Rose, 2017; Causse et al., 2011; Ke et al., 2023). Recent research papers that are focused on aviation psychophysiology support the relevance of autonomic markers in this context. HRV has been identified as a promising tool for detecting and predicting pilot mental workload, while review evidence from military aviation suggests that autonomic features are sensitive not only to flight demands, but also to recovery-related aspects of pilot mental state (Soares et al., 2025; Wang et al., 2024). Together, neuropsychophysiological stress-resilience features and cognitive task scores provide a reliable multimodal basis for the prediction of individual differences in pilot performance under startle conditions.

At the same time, multimodal physiological and behavioral data in aviation as well as machine learning (ML) methods are increasingly important tools for assessing and predicting pilot-related mental states. This is consistent with broader defense-oriented perspectives emphasizing the convergence of cognitive neuroscience and artificial intelligence as a basis for human-centered decision support, cognitive readiness, pilot selection, and resilience under uncertainty, stress, time pressure, and high cognitive load (Ćosić et al., 2023; Ćosić et al., 2026). ML models have been applied to pilot mental-state estimation, pilot behavior recognition, competency assessment in abnormal events, prediction of pilot performance, and recognition of surprise-related emotional responses using electroencephalography (EEG), electrocardiography (ECG), electrodermal activity (EDA), eye-tracking, control-input, and flight-performance data (Alreshidi et al., 2022, 2023a, b; Binias et al., 2018, 2020; Cui et al., 2021; Gumus and Saylam, 2023; Harrivel et al., 2017; Li and Lajoie, 2021; Li et al., 2022; Mishra et al., 2019; Rajendran et al., 2021; Wang et al., 2024; Zhang et al., 2022). Across this literature, regularized linear models, tree-based methods, kernel methods, neural networks, and ensemble strategies have all shown valuable results, but they differ in assumptions, interpretability, and susceptibility to overfitting, especially when samples are small and multimodal features are numerous (Heard et al., 2022; Jang et al., 2012, 2013; Kukolja et al., 2009; Šarlija et al., 2021). This makes it important to evaluate model flexibility, and if their performance gain is robust, generalizable, and scientifically interpretable.

Building on our previous work, which showed that simulator pitch-tracking performance during startle events could be predicted from a compact set of neuropsychophysiological stress-resilience features and cognitive task scores using an interpretable multiple-regression framework (Gambiraža et al., 2025), the present study compares different ML regression methods with that linear baseline. The aim was to determine whether more flexible models improve prediction of pilot performance during simulator startle events, and whether predictive performance depends more strongly on the learning algorithm or on the feature-selection strategy. To address this, models were compared under three feature-selection approaches: first, a shared domain-balanced feature set derived from the previous multiple-regression framework; second, model-specific feature selection while preserving domain balance; and third, model-specific feature selection from a broader candidate pool without requiring domain balance. This design allowed us to examine the relative influence of algorithmic complexity and feature-selection flexibility in a small-sample, multimodal prediction setting. In addition, domain-level ablation was used to assess the relative contribution of resting autonomic, startle-related, allostatic, and cognitive domains.

## 2 Materials and methods

### 2.1 Study design and participants

This study used the same dataset and overall experimental framework reported in our previous work (Gambiraža et al., 2025) and extends that study by performing a comparative evaluation of multiple ML regression methods on the same pilot-performance prediction task. The enrolled sample consisted of 17 male military pilot students from the University of Zagreb. Participants were recruited from the available cohort of military pilot students and had all undergone the nationally standardized selection process for military pilot education and training in the Republic of Croatia. This process included psychological, health, physical, and security requirements, as well as a flying evaluation lasting 5 to 7 weeks. No additional study-specific inclusion or exclusion criteria were applied before enrolment beyond membership in this available military pilot-student cohort and voluntary participation. For the present analysis, participants were retained only if complete and usable data were available for the physiological/cognitive predictor set and the simulator-performance outcome. Data from two enrolled participants were excluded because technical issues during data collection resulted in incomplete or unusable data, leaving 15 military pilot cadets for the final analysis. Participation in the study was voluntary, and all participants provided written informed consent in accordance with the guidelines of the University of Zagreb. The study was approved by the Ethics Committee of the University of Zagreb Faculty of Transport and Traffic Sciences.

### 2.2 Experimental protocol

**Figure 1:**
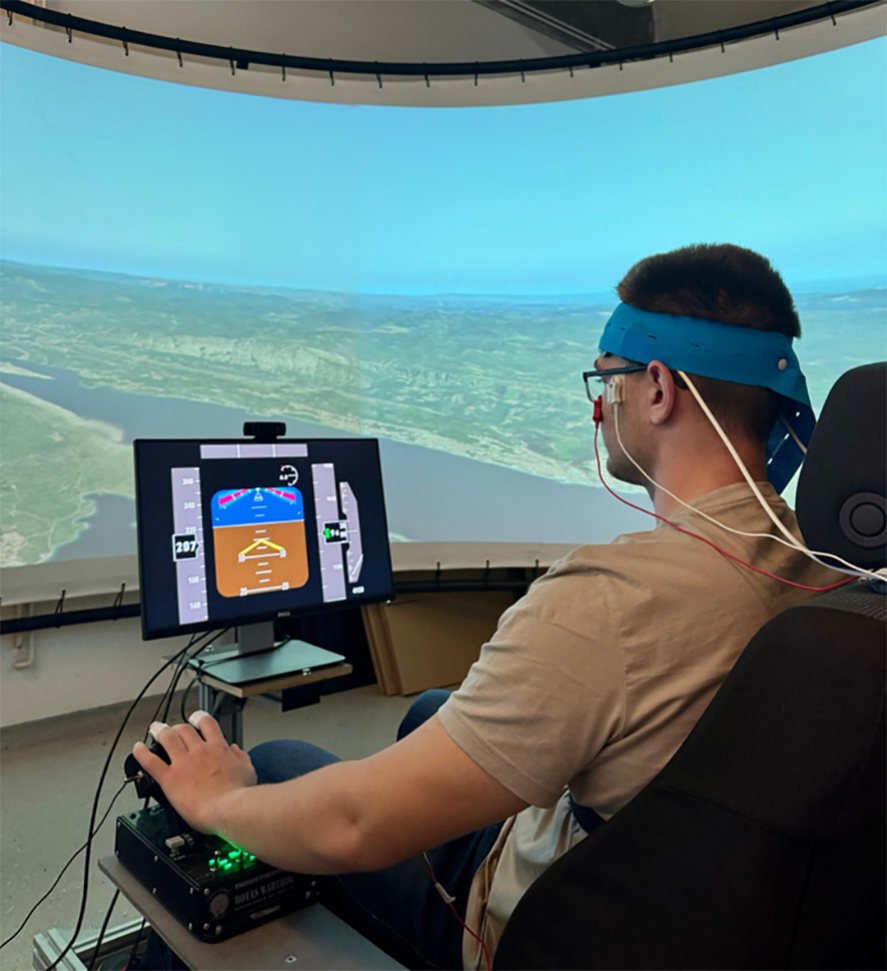
Flight-simulator setup used during the Phase 2 pitch-tracking task. Participants controlled a generic canard-configuration fighter aircraft using a flight stick, throttle lever, and rudder pedals while viewing the projected out-the-window scene and the instrument display. Unexpected wind-gust and thunder perturbations were introduced during the task to induce startle-related performance demands, and simulator performance was quantified as overall absolute pitch-tracking error. Reproduced from Gambiraža et al. (2025).

The experimental protocol consisted of two phases conducted over approximately one week. In Phase 1, participants completed a set of context-free laboratory paradigms designed to assess neuropsychophysiological features of stress resilience together with cognitive task scores. Physiological stress-resilience features were derived from a standardized acoustic startle reflex (ASR) paradigm with multimodal recording of ECG, electromyography (EMG), EDA, and respiration and were organized into three theoretical domains: resting autonomic regulation, startle reactivity and habituation, and allostatic reaction–recovery dynamics. In parallel, cognitive performance was assessed using multiple object tracking, the n-back task, the paced visual serial addition task (PVSAT), and the dual Stroop task, composed of concurrent color-word interferences and target-tone counting, forming a fourth domain of cognitive task scores. In Phase 2, participants completed a flight-simulator task involving unexpected wind-gust and thunder perturbations intended to induce startle-related performance demands. The experiment was conducted in a fixed-base research flight simulator configured to represent a generic canard-configuration fighter aircraft. The simulator consisted of two networked computers communicating via UDP: one computer executed the flight dynamics model in MATLAB/Simulink, while the second computer generated the out-the-window visual scene in X-Plane 11 and the instrument display using Sim Innovations Air Manager. The aircraft dynamics were computed using a custom six-degrees-of-freedom rigid-body flight model. Aerodynamic forces and moments were modelled using aerodynamic coefficients linearized for small angles of attack. Pilot inputs were provided using a commercial flight-control interface consisting of a flight stick, throttle lever, and rudder pedals. The external visual scene was projected using three XGA-resolution projectors onto a cylindrical screen with a radius of 2.5 m. Flight instruments were displayed on a dedicated monitor positioned in front of the pilot. The visual system provided an approximately 180° horizontal and 50° vertical field of view. The cockpit station, including the pilot seat, controls, and instrument display, was positioned in front of the projection screen. The Phase 2 simulator setup, previously described in Gambiraža et al. (2025), is shown in **Figure 1**. The prediction target in the present study was simulator performance quantified as overall absolute pitch-tracking error, defined as the deviation between the commanded pitch angle and the achieved aircraft pitch angle resulting from the pilot’s control inputs. Further procedural details are available in Gambiraža et al. (2025).

### 2.3 Data preprocessing and candidate feature set

Signal preprocessing, artifact handling, feature computation, and the full definitions of all physiological and cognitive variables were identical to those described in Gambiraža et al. (2025) and are therefore not repeated here in full. Briefly, the candidate predictor set comprised multimodal neuropsychophysiological stress-resilience features and cognitive task scores grouped into four domains. Resting autonomic features included cardiac and respiratory measures obtained during rest, such as inter-beat interval, HRV and RSA indices, and resting respiration measures. Startle-domain features captured acoustic startle magnitude, habituation, and modulation effects, including prepulse inhibition and fear-potentiated startle, derived from electromyographic and electrodermal responses. Allostatic features represented physiological reaction and recovery dynamics across stress exposure, expressed as ratios of cardiac, autonomic, and respiratory measures between baseline, reaction, and recovery phases. The cognitive domain comprised task scores from multiple object tracking, the n-back task, the paced visual serial addition task, and the dual Stroop task. The present study did not introduce new features; instead, it evaluated how different modelling strategies used the same previously derived candidate predictor space. Full feature definitions are available in Gambiraža et al. (2025).

To prevent information leakage, all fold-specific operations were performed separately within each leave-one-out cross-validation (LOOCV) iteration using only the training data. Fold-specific operations included feature standardization, correlation-based screening, feature ranking, feature subset selection, model fitting, and hyperparameter tuning where applicable. In each LOOCV iteration, one participant was held out as the test case before any of these steps were performed. When feature standardization was required, predictor variables were standardized using z-score transformation. Specifically, for each LOOCV fold, the mean and standard deviation of each predictor were estimated from the training participants only. These training-set scaling parameters were then applied to transform both the training data and the held-out participant’s predictor values. Thus, the held-out participant was not used during filtering, ranking, subset selection, estimation of standardization parameters, model fitting, or hyperparameter tuning, but only for final out-of-sample prediction.

Where correlation-based screening was applied, Pearson correlations between candidate predictors and the training-set outcome were computed within the training data of current LOOCV iteration only. These correlations were used as a descriptive feature-ranking criterion to identify predictors with the strongest fold-specific associations with simulator performance, rather than as confirmatory inferential tests. Therefore, formal assumption testing for Pearson correlation was not used as a basis for statistical inference at this screening stage. In the MRA-based feature-reduction procedure, Spearman correlations were also used together with Pearson correlations to reduce exclusive reliance on linear association ranking in line with Gambiraža et al. (2025). The highest-ranked features were retained as the candidate pool for subsequent model-specific selection procedures.

### 2.4 Comparative machine-learning framework

The primary methodological extension of the present study was a comparative evaluation of regression algorithms spanning several model families, all trained and tested on the same dataset and under the same validation protocol. For all regression models, the dependent variable was overall simulator absolute pitch-tracking error, expressed in degrees and defined as the deviation between the commanded pitch angle and the achieved aircraft pitch angle across the simulator task. Higher values indicated poorer pitch-tracking performance. The evaluated models included regularized linear methods, tree-based methods, kernel- and distance-based methods, neural approaches, and selected ensemble or auxiliary regression variants. Specifically, the comparison included ridge regression, LASSO, Bayesian ridge regression, decision tree regression, random forest regression, gradient boosting regression, support vector regression with linear, polynomial, and radial basis function kernels, k-nearest-neighbors regression, random vector functional-link networks, regression neural networks, bagging-based variants, and selected additional regression baselines such as polynomial regression.

These models were chosen to span a range of complexity and inductive assumptions, from interpretable linear estimators to more flexible nonlinear models capable of capturing higher-order relationships among multimodal physiological and cognitive predictors. The goal of the comparison was not only to identify the best-performing algorithm, but also to assess whether increased model flexibility provided a consistent practical advantage over the interpretable multiple-regression baseline in this small-sample setting.

### 2.5 Feature-selection strategies

To distinguish the effect of the learning algorithm from the effect of feature selection, three feature-selection strategies were evaluated within the same outer leave-one-out cross-validation (LOOCV) framework. In all three strategies, feature pre-selection and final feature selection were performed separately within each LOOCV training fold, not on the whole dataset. At the beginning of each LOOCV iteration, one participant was held out as the test case, and all filtering, correlation-based ranking, subset selection, model fitting, and hyperparameter tuning were performed using only the remaining training participants. The held-out participant was therefore not used at any stage of feature pre-selection, feature-combination selection, or model development, but only for final out-of-sample prediction. The three strategies differed only in how the final four-feature input set was chosen for each model and fold.

As a common starting point, all candidate predictors were first organized into four research-guided domains: resting autonomic regulation, startle reactivity and habituation, allostatic reaction–recovery dynamics, and cognitive task performance. Within each LOOCV training fold, associations between candidate predictors and simulator performance were computed using the training data only. Features were ranked according to the absolute magnitude of their association with the training-set outcome. For the MRA-based optimization procedure inherited from Gambiraža et al. (2025), both Pearson and Spearman correlations were used to reduce each domain to a smaller set of the most outcome-relevant variables before exhaustive subset evaluation. In the more data-driven model-specific procedures, Pearson correlations were used to generate a ranked pool of the 20 features with the highest absolute correlations to the training-set outcome. This top-20 pool served as the candidate feature set for subsequent subset search within the same training fold.

In the first strategy (Case 1), a fixed domain-balanced feature set was used within each cross-validation fold. Specifically, in each outer training fold, the multiple-regression optimization procedure described in Gambiraža et al. (2025) was first applied to identify a four-feature subset containing exactly one predictor from each of the four theoretical domains. Candidate domain-balanced subsets were evaluated using only the training data, and the subset that minimized training RMSE was retained as the fold-specific multiple-regression-derived feature set. That same four-feature subset was then used as input for all compared models in that fold. Thus, while the selected subset could vary across LOOCV folds, it was identical across algorithms within a given fold. This strategy provided the most controlled comparison of learning algorithms because all models were evaluated on the same compact, theory-guided input space, preserving domain balance and direct comparability with the baseline multiple-regression analysis.

In the second strategy (Case 2), feature selection was model-specific but remained domain-balanced. Within each outer training fold and for each model separately, candidate four-feature subsets were generated by selecting exactly one feature from each of the four domains. Each candidate subset was used to train the given model on the training data, and RMSE computed on the same training data was used as an apparent-error criterion for internal subset ranking. The subset with the lowest training RMSE was retained as the selected feature combination for that model in that fold. The model was then refitted on the training data using the selected four predictors and applied to the held-out participant. This strategy preserved the theoretical requirement that all four domains be represented, while allowing each algorithm to identify the domain-balanced combination that provided the best apparent fit to the available training data. To examine whether this training-RMSE-based subset-ranking procedure influenced the conclusions for flexible nonlinear models, an additional nested LOOCV sensitivity analysis was performed for selected nonlinear and flexible models. In this analysis, feature-subset evaluation was performed within an inner LOOCV loop using only the participants available in the outer training fold. The feature subset with the lowest inner-LOOCV prediction error was then selected, the model was refitted on the full outer training set using that subset, and the final prediction was made for the outer held-out participant. This analysis was applied to selected models for which the model-specific domain-balanced strategy was most relevant, including support vector regression, k-nearest-neighbours regression, random vector functional-link networks, polynomial regression, decision tree regression, random forest regression, gradient boosting regression, regression neural networks, and bagging neural networks. Nested LOOCV sensitivity analysis was applied to the model-specific domain-balanced feature-selection framework, because this framework allowed direct comparison between training-RMSE-based subset ranking and inner-LOOCV-based subset ranking while preserving the same four-domain feature structure. The analysis was not extended to the model-specific unconstrained feature-selection strategy, which involved a broader and less constrained feature-combination space.

In the third strategy (Case 3), feature selection was model-specific and unconstrained by domain balance. Within each outer training fold, all four-feature combinations drawn from the 20-feature correlation-ranked pool were evaluated separately for each model. For every candidate subset, the model was fitted on the training data and RMSE computed on the same training data was used as the apparent-error criterion for subset ranking. The four-feature subset with the lowest training RMSE was then selected, after which the model was refitted on the training data using that subset and used to predict the performance of the held-out participant. In contrast to the first two strategies, this approach did not require representation from all four theoretical domains and therefore allowed the selected subset to be determined solely by fold-specific statistical association and model-specific apparent fit.

No permutation test or leave-one-feature-out modelling procedure was used to select feature combinations in the three primary feature-selection strategies. Instead, feature combinations were selected within each LOOCV training fold according to the procedures described above: domain-balanced exhaustive subset evaluation for the MRA-derived strategy, model-specific domain-balanced subset ranking using training RMSE for the second strategy, and model-specific unconstrained subset ranking from the top-20 correlation-ranked feature pool for the third strategy. Leave-one-domain-out ablation was performed separately as a secondary explanatory analysis to evaluate the relative contribution of the four theoretical predictor domains; it was not used as a feature-selection criterion.

**Figure 2:**
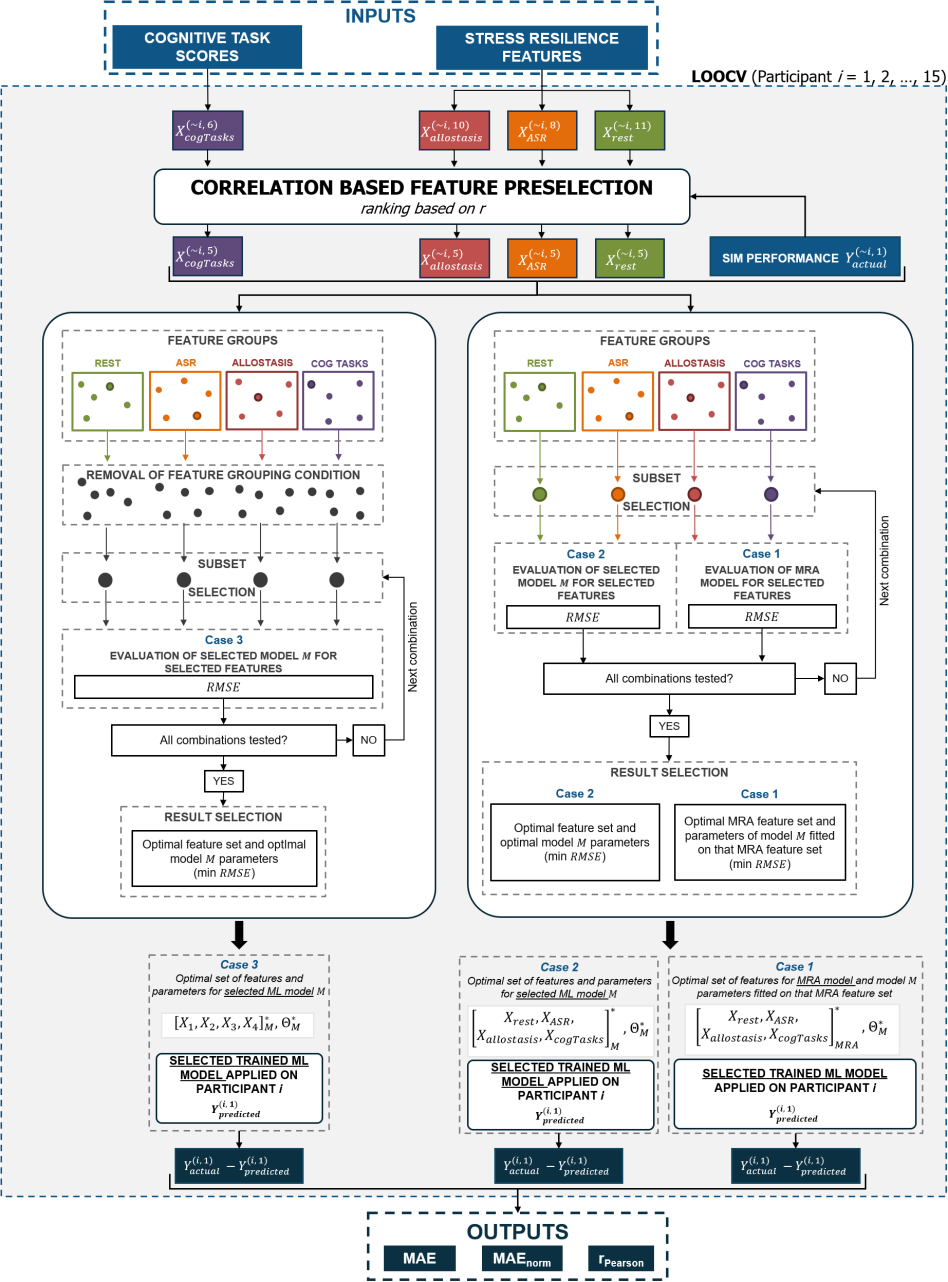
Schematic overview of the feature-selection and regression-modelling workflow within the outer leave-one-out cross-validation (LOOCV) framework. The input matrix (X) contains the candidate predictor features for all participants, including neuropsychophysiological stress-resilience features and cognitive task scores, and (Y) contains the simulator-performance outcome, defined as overall absolute pitch-tracking error. In each LOOCV iteration, one participant (i) was held out as the test case, while all feature preselection, feature ranking, subset selection, model fitting, and hyperparameter tuning were performed using only the remaining training participants. Candidate predictors were grouped into four theoretical domains: resting autonomic regulation, acoustic startle reflex characteristics, allostatic reaction–recovery dynamics, and cognitive task performance. Case 1 used a shared MRA-derived domain-balanced four-feature set for all models; Case 2 used model-specific domain-balanced feature selection; and Case 3 used model-specific unconstrained feature selection from the correlation-ranked feature pool. The symbol (Θ*_M_*) denotes the model parameters estimated during training for a given model and selected feature subset. The trained model was then applied to the held-out participant to obtain the predicted outcome ( $Y_{predicted}^{\left(i,\;1\right)}$), which was compared with the observed outcome ( $Y_{actual}^{\left(i,\;1\right)}$). Repeating this procedure across all participants produced the out-of-sample predictions used to calculate MAE, MAE_norm_, and Pearson correlation.

Together, these three strategies were designed to separate two potential sources of performance differences: the learning algorithm itself and the flexibility of the feature-selection procedure. The first strategy emphasized comparability and interpretability under a shared theory-driven input space, the second tested whether model-specific optimization within the four-domain structure improved performance, and the third examined whether less constrained data-driven feature selection yielded additional predictive benefit or instead increased overfitting risk in this small-sample setting. To improve transparency of the feature-selection results, the selected feature combinations are reported in **Supplementary Table 1**. This table lists the features selected in each LOOCV iteration for the main models under the model-specific domain-balanced feature-selection strategy, together with their corresponding theoretical domains and selection frequencies. These results allow assessment of the stability and consistency of selected predictors across LOOCV iterations and machine learning models and support interpretation of the feature-selection results from a domain-knowledge perspective. **Figure 2** provides a schematic overview of the outer LOOCV framework and illustrates how feature pre-selection, feature ranking, feature-combination selection, model fitting, and final held-out prediction were implemented in each of the three strategies.

### 2.6 Model evaluation

All models were evaluated using the same LOOCV framework across all three feature-selection strategies. In each iteration, one participant was held out as the test case and the remaining participants formed the training set. All fold-specific procedures, including any correlation-based screening, feature selection, model fitting, and hyperparameter tuning, were carried out exclusively within the training portion of each fold to prevent information leakage. The resulting model was then used to predict the held-out participant’s simulator performance.

Out-of-sample predictive performance was assessed primarily using mean absolute error (MAE) between observed and predicted pitch-tracking performance, normalized MAE (MAE_norm_), and the Pearson correlation between predicted and observed performance. MAE was expressed in degrees of pitch-tracking error, which required inverse z-score transformation of predictive model output using training set mean and standard deviation. Similarly, MAE_norm_ was computed as mean absolute error between predicted and z-score-transformed observed pitch tracking error. Training RMSE was used only as an internal criterion during feature-selection procedures and was not interpreted as a primary indicator of out-of-sample generalization performance. For the main models, uncertainty in out-of-sample performance was estimated using bootstrap resampling of participant-level LOOCV predictions. Specifically, 95% confidence intervals for MAE, MAE_norm_, and Pearson correlation were computed using 1,000 bootstrap resamples. To improve reproducibility, the main model hyperparameters, tuning ranges or final selected values, software platform, package/toolbox versions, and random seed used for the comparative modelling analyses are reported in **Supplementary Table 2**.

### 2.7 Domain-level ablation analysis

To assess the relative contribution of the four theoretical predictor domains, a leave-one-domain-out ablation analysis was conducted as a secondary explanatory analysis for a subset of models. Because this analysis requires a reference configuration containing one predictor from each theoretical domain, it was performed under the domain-balanced feature-selection strategy that showed stronger out-of-sample performance in the comparative analysis. Within each cross-validation fold, the full four-feature model containing one predictor from each domain served as the reference configuration. The feature belonging to one domain was then removed, the model was retrained using the remaining features, and predictive performance on the held-out participant was recomputed. The contribution of a domain was quantified as the degradation in predictive performance relative to the full model, primarily in terms of the increase in MAE. Positive degradation values indicated that omission of the domain worsened prediction. This analysis was used to interpret the relative influence of resting autonomic, startle-related, allostatic, and cognitive information on pilot-performance prediction, rather than as a separate model-selection criterion.

## 3 Results

### 3.1 Overall comparative model performance

**Table 1** summarizes the out-of-sample predictive performance of the baseline multiple-regression model and all evaluated ML models under the fixed domain-balanced, model-specific domain-balanced, and model-specific unconstrained feature-selection strategies. Overall, predictive performance depended strongly on the feature-selection strategy in addition to the learning algorithm itself. Across models, the fixed domain-balanced feature strategy yielded the strongest and most stable results, whereas the more flexible model-specific strategies, particularly the model-specific unconstrained feature strategy, generally reduced predictive performance. This pattern is consistent with the broader comparison showing that stable, theory-guided feature sets supported more robust leave-one-out generalization than model-specific combinatorial feature search in this small-sample setting.

**Table 1:** Out-of-sample predictive performance of the baseline multiple-regression model and all evaluated ML models across feature-selection strategies.

Model	Fixed domain-balanced feature strategy		Model-specific domain-balanced feature strategy		Model-specific unconstrained feature strategy	
	MAEnorm	r	MAEnorm	r	MAEnorm	r
MRA	0.6143	0.7039	0.6143	0.7039	1.4301	-0.1552
Ridge	0.6080	0.6929	0.6080	0.6929	1.2830	-0.1246
LASSO	0.6905	0.6221	0.7000	0.5967	0.8157	0.4038
Decision Tree	1.1232	-0.0779	1.3672	-0.1782	0.9894	0.1586
Random Forest	0.8600	0.2505	0.9643	0.0347	0.9419	-0.0923
GBM	0.6108	0.6850	1.1285	0.1950	1.3049	-0.3475
SVR linear	0.6279	0.7120	0.7230	0.6057	0.8760	0.3515
SVR poly	0.9034	0.5336	3.5071	-0.1756	1.6635	0.3349
SVR rbf	0.8647	0.3951	1.0454	-0.0034	1.0652	-0.0945
kNN (best k)	0.8506	0.7400	0.8320	0.2005	0.7957	0.5083
RVFLN	0.6349	0.6679	1.2364	-0.6234	0.9176	0.3274
RegNN	**0.4994**	**0.7457**	1.7729	-0.6950	1.0823	0.1213
Polynomial	0.6143	0.7039	1.0354	0.1709	1.5508	0.2283
Bayesian Ridge	0.6133	0.7028	0.6133	0.7028	1.4103	-0.1516
Bagging NN	0.5518	0.6657	0.8009	0.2747	**0.6678**	**0.5270**

The fixed domain-balanced feature strategy used the same fold-specific four-feature subset for all models, with one predictor selected from each theoretical domain. The model-specific domain-balanced feature strategy optimized a four-feature subset separately for each model while preserving the one-predictor-per-domain rule. The model-specific unconstrained feature strategy optimized a four-feature subset separately for each model from a top-20 correlation-ranked feature pool without requiring domain balance. Reported metrics are normalized mean absolute error (MAE_norm_) and Pearson correlation between predicted and observed simulator performance (r). For k-nearest-neighbors regression, the fixed domain-balanced feature strategy used the baseline k = 10, whereas the model-specific domain-balanced and model-specific unconstrained strategies report the best-performing k values among those tested (k = 5 and k = 7, respectively). For polynomial regression, the polynomial degree was fixed at 2.

Bold values indicate the best-performing values within each comparison, i.e., the lowest error metric and/or the highest Pearson correlation.

Under the fixed domain-balanced feature strategy, the best-performing model was the regression neural network, which achieved the lowest normalized mean absolute error and the highest correlation between predicted and observed simulator performance (MAE_norm_ = 0.4994, r = 0.7457). The bagging neural network also performed well (MAE_norm_ = 0.5518, r = 0.6657). However, the interpretable baseline multiple-regression model remained highly competitive (MAE_norm_ = 0.6143, r = 0.7039), with performance closely matched by ridge regression (0.6080, 0.6929), Bayesian ridge regression (0.6133, 0.7028), polynomial regression of degree 2 (0.6143, 0.7039), and linear support vector regression (0.6279, 0.7120). These findings indicate that although some nonlinear models achieved slightly better predictive accuracy, the practical advantage over the baseline was modest.

In contrast, performance under the model-specific domain-balanced feature strategy was generally weaker and less consistent. Among the models evaluated under this strategy, ridge regression produced the lowest normalized MAE (0.6080), while the baseline multiple-regression model and Bayesian ridge regression remained close in performance. Several nonlinear and tree-based models deteriorated substantially under this per-model domain-balanced optimization, including the regression neural network, random vector functional-link network, and polynomial support vector regression.

The model-specific unconstrained feature strategy yielded the weakest overall results. In this setting, Bagging NN achieved the lowest normalized MAE (0.6678) and one of the higher correlations (r = 0.5270), while k-nearest-neighbors regression also retained moderate correlation (r = 0.5083). However, most models showed marked deterioration relative to the fixed domain-balanced feature strategy, and the baseline multiple-regression model performed particularly poorly (MAE_norm_ = 1.4301, r = -0.1552). Taken together, these findings indicate that increasing flexibility in feature selection did not improve out-of-sample generalization in this small-sample setting and, in many cases, degraded it.

### 3.2 Results under the fixed domain-balanced feature configuration

Because the fixed domain-balanced feature strategy provided the most controlled comparison of ML algorithms by evaluating all models on the same fold-specific four-feature set, this configuration provides the clearest basis for algorithm-level interpretation. **Table 2** reports the out-of-sample performance of the main models under this configuration, including MAE, MAE_norm_, Pearson correlation, and 95% bootstrap confidence intervals. **Figure 2** complements these numerical results by showing predicted-versus-observed simulator performance for the same models.

**Table 2:** Out-of-sample predictive performance of the main models under the fixed MRA-based feature strategy.

Model	MAE	95% CI MAE	MAEnorm	95% CI MAEnorm	Pearson r	95% CI Pearson r
MRA	2.55	[1.54, 3.63]	0.212	[0.128, 0.302]	0.655	[0.376, 0.864]
Ridge	2.51	[1.58, 3.51]	0.209	[0.132, 0.292]	0.634	[0.356, 0.878]
Bayesian Ridge	2.54	[1.55, 3.71]	0.211	[0.129, 0.308]	0.653	[0.374, 0.865]
SVR linear	2.60	[1.73, 3.53]	0.216	[0.144, 0.293]	0.660	[0.406, 0.860]
RegNN	2.11	[1.08, 3.38]	0.175	[0.090, 0.281]	0.691	[0.326, 0.925]
Bagging NN	2.37	[1.21, 3.64]	0.197	[0.101, 0.303]	0.571	[0.212, 0.879]

Values are point estimates with 95% confidence intervals shown in brackets. Confidence intervals were estimated using 1,000 bootstrap resamples of participant-level LOOCV predictions.

Within this comparison, the regression neural network achieved the lowest prediction error (MAE = 2.11°, MAE_norm_ = 0.175) and the highest Pearson correlation among the main models (r = 0.691), followed by the bagging neural network in terms of prediction error (MAE = 2.37°, MAE_norm_ = 0.197). However, the interpretable baseline multiple-regression model remained highly competitive (MAE = 2.55°, MAE_norm_ = 0.212, r = 0.655), with closely comparable performance from ridge regression, Bayesian ridge regression, and linear support vector regression. The bootstrap confidence intervals were relatively wide and partly overlapping across models, as expected given the small sample size, indicating that differences among the main models should be interpreted cautiously.

The magnitude of the performance differences under this configuration was relatively small compared with the differences observed across feature-selection strategies. In particular, the improvement of the best-performing regression neural network over the baseline multiple-regression model was modest, whereas the baseline retained the advantage of straightforward interpretability. Notably, several more flexible methods, including tree-based models and nonlinear-kernel support vector regression, did not outperform the baseline under this shared feature configuration. This pattern suggests that when all models operate on the same compact and theory-guided multimodal inputs, algorithmic complexity alone does not guarantee better generalization.

**Figure 3:**
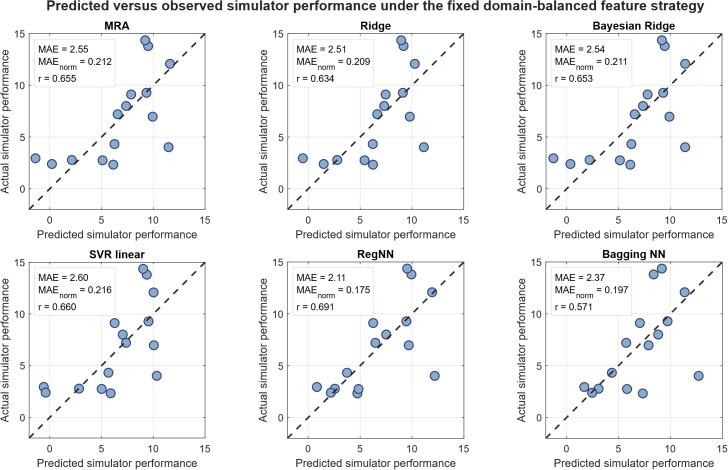
Predicted versus observed simulator performance under the fixed domain-balanced feature strategy. Scatter plots show out-of-sample LOOCV predictions for the six main models using the fixed MRA-selected feature sets. The x-axis shows predicted simulator performance, and the y-axis shows observed simulator performance. The dashed diagonal line represents perfect prediction. Insets show MAE, normalized MAE (MAE_norm_), and Pearson correlation coefficient (r) for each model. Residual plots for the same models are provided in **Supplementary Figure 1**.

The predicted-versus-observed plots in **Figure 3** show broadly similar prediction patterns across the main models and provide a visual check of whether performance was driven by a small number of influential observations. Residual plots for the same models are provided in **Supplementary Figure 1** to support inspection of prediction bias and potential outliers.

### 3.3 Influence of feature-selection strategy

Comparison across the three feature-selection strategies showed that feature-selection design had a strong influence on predictive performance. For most models, the fixed domain-balanced feature strategy produced better or more stable results than either model-specific strategy. The model-specific domain-balanced feature strategy, despite preserving the one-predictor-per-domain constraint, did not improve out-of-sample performance overall and often led to deterioration relative to the shared feature setting. The model-specific unconstrained feature strategy, which removed the domain-balance constraint and selected features from a correlation-ranked pool, produced the weakest overall pattern and the largest number of poorly performing models.

This effect was also evident at the group level. As summarized in **Table 3**, the fixed domain-balanced feature strategy yielded the lowest mean normalized MAE and the highest mean Pearson correlation across all evaluated models. In contrast, both model-specific strategies showed substantially poorer average performance, with the model-specific domain-balanced strategy producing the highest mean normalized MAE and the lowest mean correlation overall. These aggregate results reinforce the conclusion that stable, theory-guided feature selection provided a more robust basis for out-of-sample generalization than more flexible model-specific optimization in this small-sample setting.

**Table 3:** Mean predictive performance across all evaluated models under the three feature-selection strategies.

Feature-selection strategy	Mean MAEnorm	Mean r
Fixed domain-balanced feature strategy	**0.7184**	**0.5741**
Model-specific domain-balanced feature strategy	1.1666	0.1281
Model-specific unconstrained feature strategy	1.0974	0.1536

Reported values represent the mean normalized mean absolute error (MAE_norm_) and mean Pearson correlation (r) across models.

Bold values indicate the best-performing values within each comparison, i.e., the lowest error metric and/or the highest Pearson correlation.

This pattern was particularly clear for models that performed strongly under the fixed domain-balanced feature strategy but deteriorated substantially under more flexible feature-selection settings. The regression neural network is the clearest example: it was the best-performing model under the fixed shared-feature configuration but showed large reductions in performance under both model-specific strategies. A similar pattern was observed for gradient boosting, Bayesian ridge regression, and the baseline multiple-regression model. Overall, these findings indicate that in this small-sample multimodal dataset, generalization depended more strongly on feature-set stability and theoretical structure than on model flexibility alone.

To further examine the influence of the feature-subset ranking criterion, a nested LOOCV sensitivity analysis was performed for selected nonlinear and flexible models under the model-specific domain-balanced feature-selection framework. Overall, this analysis did not change the main interpretation. Linear SVR showed the strongest performance among the evaluated nested-LOOCV models, with MAE = 2.74°, normalized-scale MAE = 0.6607, and r = 0.6521. This performance was better than the corresponding model-specific domain-balanced SVR result obtained using training-RMSE-based subset ranking, but it did not outperform the strongest models under the fixed domain-balanced feature strategy. The selected features for linear SVR were also highly similar to the fixed domain-balanced configuration, with EMG habituation (HabitEMG) and IBI recovery selected in all outer folds, and dual Stroop performance selected in most folds.

The regression neural network achieved MAE = 2.93°, MAE_norm_ = 0.7125, and r = 0.3852 under nested LOOCV, which was weaker than its performance under the fixed domain-balanced feature strategy. Bagging neural networks showed a similar pattern, with MAE = 2.93°, MAE_norm_ = 0.7202, and r = 0.3065 under nested LOOCV. The remaining nonlinear or flexible models, including polynomial-kernel and Gaussian-kernel support vector regression, decision tree, random forest, gradient boosting, k-nearest-neighbors regression, random vector functional-link networks, and polynomial regression, also did not show a consistent generalization advantage under nested LOOCV. The nested LOOCV sensitivity analysis was therefore kept focused on the domain-balanced framework, because the main analysis already showed weaker performance for the unconstrained feature-selection framework and the nested domain-balanced analysis did not indicate a general advantage over the fixed domain-balanced strategy.

### 3.4 Domain-level ablation analysis

**Figure 4:**
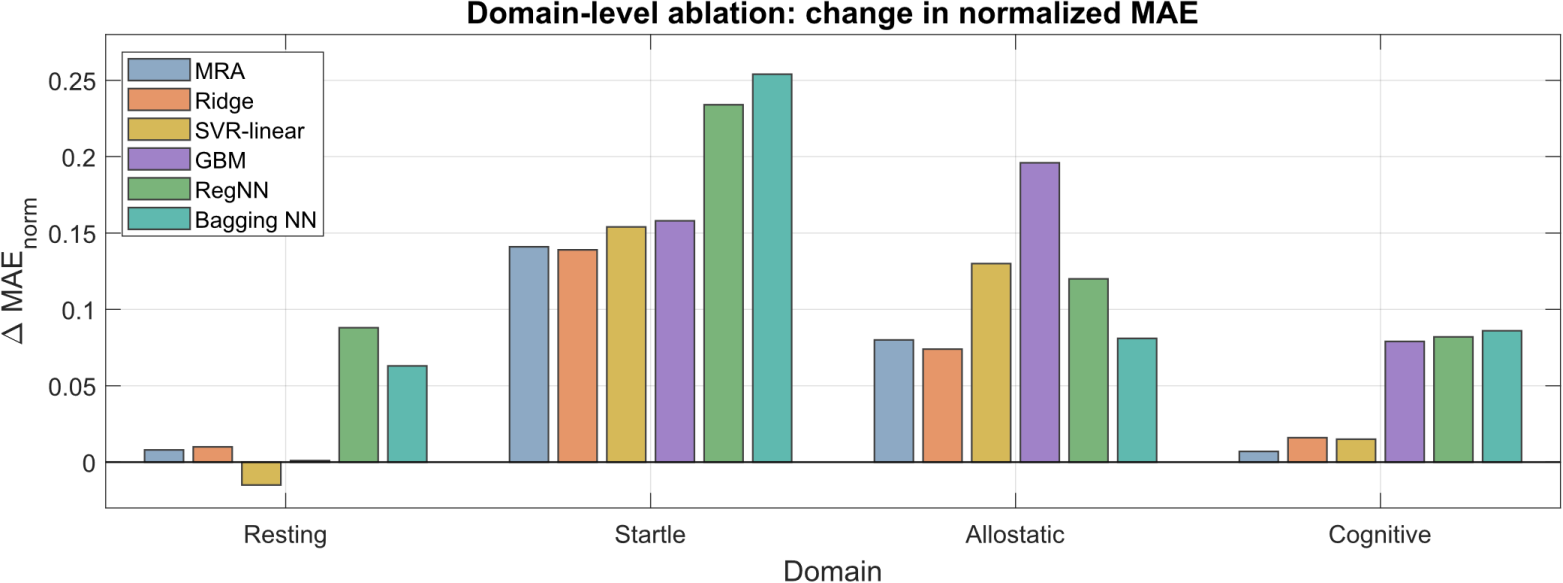
Domain-level ablation results under the fixed domain-balanced feature strategy. Bars show the change in normalized mean absolute error (ΔMAE_norm_) relative to the full four-feature model after removal of the feature representing each theoretical domain for the baseline multiple-regression model (MRA), ridge regression, linear support vector regression (SVR-linear), gradient boosting (GBM), the regression neural network (RegNN), and Bagging NN.

A leave-one-domain-out ablation analysis was conducted as a secondary explanatory analysis under the fixed domain-balanced feature strategy to examine the relative contribution of the four theoretical predictor domains. **Figure 4** summarizes the resulting changes in normalized MAE relative to the corresponding full four-feature model for the selected representative models. This analysis was performed for the baseline multiple-regression model, ridge regression, linear support vector regression, gradient boosting, the regression neural network, and the bagging neural network.

Across all models, removal of the startle-domain feature produced the largest increase in prediction error, indicating that this domain contributed most strongly to pilot-performance prediction. The increase in normalized MAE after removal of the startle feature ranged from 0.139 to 0.254, corresponding to an increase of approximately 1.7° to 3.1° in mean absolute pitch-tracking error. The strongest degradation was observed for Bagging NN, followed by the regression neural network and gradient boosting. Removal of the allostatic-domain feature also consistently worsened predictive performance across models, although to a lesser extent than removal of the startle-domain feature. The increase in normalized MAE ranged from 0.074 to 0.196, corresponding to approximately 0.9° to 2.4° on the original error scale. This pattern suggests that physiological reaction and recovery dynamics provided an important complementary contribution to prediction across both linear and nonlinear models.

In contrast, removal of the resting autonomic or cognitive-domain feature generally produced smaller changes in predictive performance, particularly for the linear models. For the baseline multiple-regression model and ridge regression, omission of either of these domains resulted in only minimal increases in error. However, for the neural models, especially the regression neural network and Bagging NN, removal of resting or cognitive information produced somewhat larger degradations, reaching approximately 1° on the original error scale. One exception was linear support vector regression, for which removal of the resting-domain feature resulted in a small reduction in error. Overall, the ablation results indicate that the strongest predictive contribution within the domain-balanced four-feature configuration arose from startle-related and allostatic information, while resting autonomic and cognitive domains made smaller but model-dependent contributions. These findings complement the comparative model results by showing that the relative importance of predictor domains was not uniform, and that the most influential information for pilot-performance prediction was associated primarily with acute defensive reactivity and physiological recovery dynamics.

## 4 Discussion

The present study extended our previous regression-based analysis of pilot performance during simulator startle events by comparing multiple ML regression methods on the same multimodal dataset and by explicitly examining the effect of feature-selection strategy on out-of-sample generalization. Three main findings emerge. First, several ML models matched or modestly exceeded the predictive performance of the baseline multiple-regression model when trained on the same compact multimodal input space. Second, the strongest determinant of robust out-of-sample performance was not model flexibility alone, but the stability and structure of the selected feature set. Third, domain-level ablation indicated that startle-related and allostatic features contributed most strongly to prediction, whereas resting autonomic and cognitive domains made smaller but still meaningful contributions. Together, these findings suggest that, in small and heterogeneous aerospace datasets, stable theory-guided feature representations may be more important than increasing algorithmic complexity.

### 4.1 Comparative model performance and the role of interpretability

A central question of this study was whether more flexible nonlinear ML methods provide a meaningful advantage over an interpretable regression baseline when predicting startle-related simulator performance from compact, theory-driven inputs. The answer appears to be nuanced. Under the fixed domain-balanced feature strategy, the regression neural network achieved the best predictive performance, and the bagging neural network also performed strongly. However, the practical gain over the baseline multiple-regression model was modest. At the same time, the baseline remained highly competitive and was closely matched by ridge, Bayesian ridge, and linear support vector regression. This pattern suggests that a large proportion of the predictive structure in the selected four-feature input space is already captured by relatively simple models.

In the present setting, the best nonlinear model did not produce a large step-change in performance, and several flexible model families, particularly tree-based methods and nonlinear-kernel support vector regression, failed to outperform the baseline. This is consistent with the classical bias–variance argument that, in very small samples, lower-capacity models often generalize more reliably than more flexible ones unless there is strong evidence of stable nonlinear structure in the data (Hastie et al., 2009; James et al., 2021; Kuhn & Johnson, 2013). The current findings therefore favour a cautious interpretation: modest nonlinear gains are possible, but they do not eliminate the practical value of interpretable regression models in pilot-performance prediction.

Interpretability is particularly important in this application. Our previous study (Gambiraža et al., 2025) showed that a compact set of stress-resilience and cognitive predictors can predict simulator performance under startle. The present study extends that result by showing that a neural model can slightly improve accuracy under the most stable feature configuration, but the explanatory insight still comes primarily from the structured regression baseline and the domain-level analyses. This distinction matters for aerospace applications, where the scientific and operational usefulness of a model depends not only on predictive accuracy but also on whether the selected predictors can be linked back to interpretable physiological and cognitive constructs relevant to assessment, training, and monitoring (Ćosić et al., 2019b; Šarlija et al., 2021; Gambiraža et al., 2025).

### 4.2 Feature-selection was more important than algorithmic flexibility

The main methodological result of this study is that feature-selection strategy had a stronger influence on performance than model class. Across the full comparison, the fixed domain-balanced feature strategy consistently outperformed the model-specific alternatives on average. By contrast, the model-specific domain-balanced and unconstrained strategies generally reduced performance, even though they offered more flexibility and, in principle, greater opportunity to tailor the predictor set to each model. This indicates that, in the present small-sample LOOCV setting, additional flexibility in feature search did not improve generalization and instead increased instability.

This finding has broader methodological significance. In multimodal psychophysiological datasets, especially those with small numbers of participants and correlated candidate features, model-specific feature optimization can easily overfit fold-specific noise. That risk becomes particularly pronounced when feature selection and model fitting are both flexible and are repeated within many possible subset combinations. The current results suggest that once a stable and theory-consistent feature representation is identified, model choice becomes comparatively less influential, whereas unstable feature search can degrade performance even for otherwise strong algorithms. This interpretation is consistent with general methodological guidance from predictive modelling, which emphasizes that data scarcity and high search flexibility increase optimism bias and reduce out-of-sample reliability (Hastie et al., 2009; James et al., 2021; Kuhn & Johnson, 2013).

The behaviour of the regression neural network illustrates this point particularly clearly. It achieved the best overall performance under the fixed domain-balanced strategy, but deteriorated sharply under both model-specific strategies, especially the model-specific domain-balanced setting. This suggests that the model itself was not inherently unstable; rather, it benefited strongly from being trained on a compact and stable input representation and became vulnerable when feature selection varied across folds. The same general tendency was observed for other nonlinear methods. Thus, the present findings do not argue against nonlinear modelling per se, but they do indicate that in small-*N* pilot datasets, the benefit of nonlinear estimators depends critically on having a robust and physiologically meaningful feature space.

The additional nested LOOCV sensitivity analysis supports the same interpretation. When selected nonlinear and flexible models were re-evaluated using inner-LOOCV feature-subset selection within the domain-balanced framework, the results did not show a general improvement over the fixed domain-balanced feature strategy. Linear SVR retained moderate predictive performance, but its selected feature configuration was highly similar to the fixed domain-balanced feature set, particularly through repeated selection of EMG habituation, IBI recovery, and dual Stroop performance. More flexible nonlinear models, including the regression neural network, tree-based models, RVFL, kNN, polynomial regression, and nonlinear-kernel SVR variants, did not consistently benefit from nested feature-subset optimization. This indicates that the limited performance of the model-specific strategies was not simply an artifact of using training RMSE for subset ranking, but more likely reflected the instability of flexible feature selection in a very small multimodal dataset.

One methodological consideration is that the fixed domain-balanced feature strategy may have been somewhat favourable to the baseline multiple-regression model and related linear estimators, because the shared four-feature subset in each fold was selected using the multiple-regression optimization procedure from Gambiraža et al. (2025), with subset choice based on minimization of training RMSE. Thus, the strongest comparison setting in the present study was not fully neutral with respect to model class. At the same time, this strategy provided the most controlled algorithm-level comparison because all models were evaluated on the same compact, theory-guided feature set within each fold. The poorer performance of the more flexible model-specific strategies further suggests that, in this small-sample setting, the advantage of the fixed strategy was not simply due to favouring the baseline, but also to the stabilizing effect of a shared and physiologically structured input space.

### 4.3 Interpretation of domain-level contributions

The domain-level ablation analysis provides an additional interpretive layer by showing that the four predictor domains did not contribute equally to prediction. Across representative models, removal of the startle-domain feature produced the largest increase in error, and removal of the allostatic-domain feature also caused consistent performance degradation. These results indicate that startle-related and reaction–recovery dynamics carried the strongest predictive signal in the compact four-feature models. Resting autonomic and cognitive domains generally contributed less, although they were not negligible and their influence varied across model families.

This pattern is conceptually plausible. Startle-domain features capture the intensity and adaptation of defensive responding, while allostatic features reflect how effectively physiological systems recover after perturbation. Both are directly relevant to resilience under unexpected threat. In contrast, resting autonomic features may provide broader information about baseline regulatory capacity, and cognitive task scores may represent more general executive and attentional abilities that support adaptation but are less proximal to the immediate physiological disruption caused by startle. The current findings therefore support the view that pilot vulnerability during sudden perturbation is shaped most strongly by the dynamics of defensive reactivity and recovery, with cognitive capacity and baseline regulation providing additional context rather than dominant predictive information. This interpretation is consistent with prior work linking stress-resilience physiology to task performance and with the broader stress-resilience framework used to construct the predictor domains (Ćosić et al., 2019b; Šarlija et al., 2021; Gambiraža et al., 2025).

At the same time, these domain-level findings should be interpreted carefully. The ablation analysis was conducted as a secondary explanatory analysis under the better-performing domain-balanced strategy, and therefore its results are best viewed as explanatory rather than confirmatory. Even so, the convergence of the ablation results across several models strengthens confidence that startle and allostatic processes are central components of the predictive signal rather than artifacts of any one model class.

### 4.4 Relation to prior aviation and machine-learning literature

The present findings fit within two overlapping strands of literature. The first concerns aviation human factors and startle-related performance. Prior studies have shown that startle and surprise can impair attention, information processing, and operational performance in flight-related tasks, and that recovery from such events is highly variable across individuals (Landman et al., 2017; Kinney and O’Hare, 2020; Diarra et al., 2023; Deniel et al., 2023; Duchevet et al., 2025; Vlaskamp et al., 2025). The current results extend that literature by showing that individual differences in context-free neuropsychophysiological stress-resilience features and cognitive task scores can be used not only to explain but also to predict startle-related performance differences.

The second strand concerns machine learning for pilot-state and performance prediction. Many recent studies have applied machine-learning methods to pilot workload estimation, cognitive-state classification, surprise detection, and behaviour recognition from EEG, ECG, eye tracking, and related modalities (Harrivel et al., 2017; Binias et al., 2018, 2020; Li et al., 2022; Alreshidi et al., 2023b, 2024). Much of that work focuses on classification or larger-scale state detection problems, where flexible models can outperform simpler baselines. The present study shows that this advantage does not necessarily transfer to very small-*N* regression problems with heavily preselected and domain-balanced multimodal features. In such settings, the main methodological challenge may be identifying a stable, theory-grounded predictor representation rather than searching for ever more complex estimators.

### 4.5 Operational implications for next-generation air combat

The present study has several implications for the broader research topic of optimizing pilot performance in next-generation air combat. Modern fighter operations expose pilots to abrupt perturbations, high workload, dense information streams, and compressed time for response. In such environments, the ability to anticipate which individuals are more likely to maintain performance under startle has potential relevance for selection, training, and readiness monitoring. The current findings suggest that compact multimodal measures of stress resilience and cognition may contribute to such efforts, particularly when used in a theory-guided and explainable modelling framework.

At a practical level, the results do not support relying on black-box model complexity as the primary path forward. Instead, they point toward a more conservative translational strategy: first identify robust, interpretable, and domain-consistent markers of stress resilience, then evaluate whether modest nonlinear models add incremental value on top of that stable representation. For applications such as pilot screening, resilience-oriented training, or adaptive monitoring, this may be more realistic and defensible than deploying highly flexible models trained on unstable feature sets from very small samples.

### 4.6 Limitations

Several limitations should be acknowledged. First, the sample size was small (N = 15), which constrains statistical power, increases sensitivity to fold-wise variation under LOOCV, and limits the extent to which conclusions can be generalized beyond the present cohort. In addition, the sample included only male military pilot cadets. Therefore, the present study could not evaluate sex-related differences in neuropsychophysiological stress-resilience features, cognitive task performance, startle responses, or simulator pitch-tracking performance. Such differences may be relevant, because autonomic regulation, stress reactivity, and recovery dynamics can vary with sex-related physiological factors, including hormonal influences and menstrual-cycle phase (Kofler et al., 2001; Armbruster et al., 2014). Future studies should therefore include female pilots and larger mixed-sex samples to determine whether the predictive relationships observed here generalize across sex. Second, the study used military pilot cadets rather than experienced operational fighter pilots, so the findings may not fully reflect the adaptation, compensatory strategies, or stress profiles of more experienced aircrew. Third, the outcome measure was a specific simulator-based pitch-tracking metric rather than a broader multidimensional index of operational flight performance. Fourth, the comparative framework focused on compact four-feature models for reasons of interpretability and robustness in a small-sample setting. Although a less constrained model-specific strategy based on a top-20 correlation-ranked pool was also examined, the study did not perform an unrestricted search over larger feature subsets, and some higher-dimensional nonlinear structure may therefore have remained unexploited. In addition, an additional nested LOOCV sensitivity analysis was performed for selected nonlinear and flexible models under the model-specific domain-balanced feature-selection framework. However, the small sample size still limits the stability of feature-subset optimization, particularly when many candidate feature combinations and model families are evaluated. The nested LOOCV approach applied to non-linear models showed no improvements relative to their performance metrics shown in **Table 1** for model-specific domain-balanced feature strategy. Accordingly, the nested analysis was not extended to the unconstrained feature-selection strategy, which exhibited similar performance results in **Table 1** despite having a broader and less constrained feature-combination space. Therefore, the feature-selection results should still be interpreted cautiously and should be validated in larger independent datasets. The study also did not include a shuffled-outcome or permutation-based null-model analysis across the full modelling pipeline. Therefore, the present results should be interpreted as preliminary comparative evidence within a small-sample dataset rather than as definitive chance-corrected evidence of predictive performance. Future studies with larger datasets or a smaller set of prespecified models should include permutation-based null-model testing to evaluate whether observed performance exceeds chance-level prediction. Finally, the domain-level ablation analysis was conducted as a secondary explanatory analysis under the better-performing domain-balanced strategy and should therefore be interpreted cautiously.

## 5 Conclusion

In summary, the present study suggests that pilot simulator performance during startle events can be predicted from compact multimodal neuropsychophysiological stress-resilience features and cognitive task scores using both interpretable regression and ML models. Although the regression neural network achieved the best performance under the fixed domain-balanced feature strategy, its advantage over the multiple-regression baseline was modest. The more important finding was methodological: across the main comparison and additional nested LOOCV sensitivity analysis, out-of-sample performance depended more strongly on stable, theory-guided feature selection than on model flexibility alone. Domain-level ablation further indicated that startle-related and allostatic information contributed most strongly to prediction. Although larger-sample validation and chance-level benchmarking remain necessary before such models can be interpreted as robust predictors for operational use, the present findings support the potential value of compact, explainable, and physiologically grounded modelling strategies for small aerospace datasets and provide a basis for future work on pilot assessment, resilience-oriented training, and human-centred performance optimization in advanced air combat systems.

## Data Availability

The dataset generated and analyzed during the current study is not publicly available and is not available upon request because access is restricted by project-specific confidentiality obligations and data-sharing limitations established in the research agreement. Requests to access the datasets should be directed to mate.gambiraza@fer.unizg.hr.
