# Supplementary figures and images for: Comparative analysis of machine-learning methods for prediction of pilot performance during startle events from neuropsychophysiological features of stress resilience and cognitive task scores
Source: Front Physiol. 2026 Jul 10;17:1879388. doi: 10.3389/fphys.2026.1879388 (PMC13395618; doi:10.3389/fphys.2026.1879388)

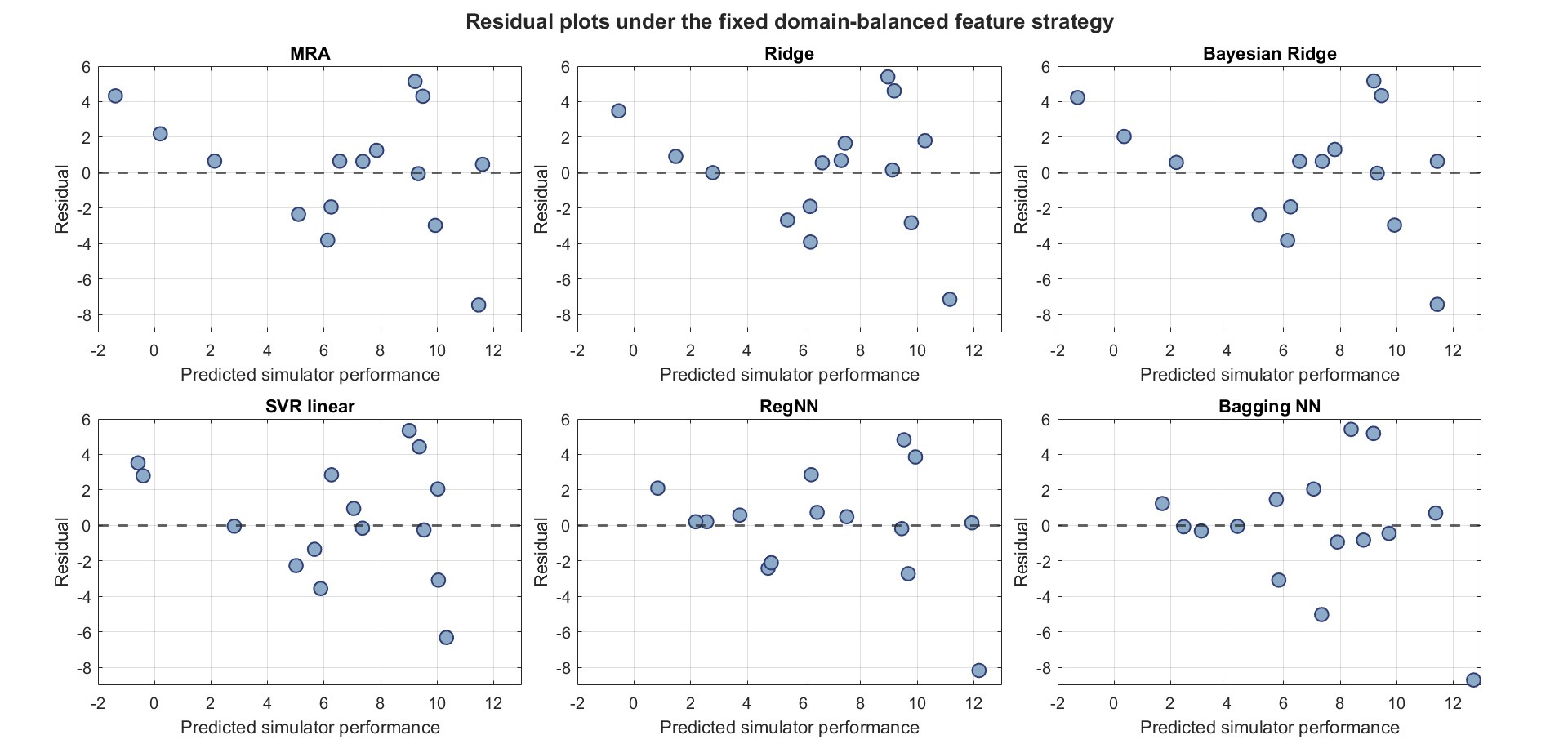

Supplement: Supplementary file 1 [file Image1.png]
